# Multi-omics analyses on *Kandelia obovata* reveal its response to transplanting and genetic differentiation among populations

**DOI:** 10.1186/s12870-021-03123-1

**Published:** 2021-07-19

**Authors:** Yuze Zhao, Yifan Zhong, Congting Ye, Pingping Liang, Xiaobao Pan, Yuan-Ye Zhang, Yihui Zhang, Yingjia Shen

**Affiliations:** 1 Key Laboratory of the Ministry of E, ducation for Coastal and Wetland Ecosystems, College of the Environment and Ecology, Xiamen University, Xiamen, 361102 Fujian China; 2 Key Laboratory of Tropical Translational Medicine of Ministry of Education, School of Tropical Medicine and Laboratory Medicine, Hainan Medical University, Haikou, 571199 China

**Keywords:** DNA methylation, Genetic differentiation, *Kandelia obovata*, Latitudinal gradient, Population reciprocal transplant, Transcriptome

## Abstract

**Background:**

Restoration through planting is the dominant strategy to conserve mangrove ecosystems. However, many of the plantations fail to survive. Site and seeding selection matters for planting.

The process of afforestation, where individuals were planted in a novel environment, is essentially human-controlled transplanting events. Trying to deepen and expand the understanding of the effects of transplanting on plants, we have performed a seven-year-long reciprocal transplant experiment on *Kandelia obovata* along a latitudinal gradient.

**Results:**

Combined phenotypic analyses and next-generation sequencing, we found phenotypic discrepancies among individuals from different populations in the common garden and genetic differentiation among populations. The central population with abundant genetic diversity and high phenotypic plasticity had a wide plantable range. But its biomass was reduced after being transferred to other latitudes. The suppressed expression of lignin biosynthesis genes revealed by RNA-seq was responsible for the biomass reduction. Moreover, using whole-genome bisulfite sequencing, we observed modification of DNA methylation in MADS-box genes that involved in the regulation of flowering time, which might contribute to the adaptation to new environments.

**Conclusions:**

Taking advantage of classical ecological experiments as well as multi-omics analyses, our work observed morphology differences and genetic differentiation among different populations of *K. obovata*, offering scientific advice for the development of restoration strategy with long-term efficacy, also explored phenotypic, transcript, and epigenetic responses of plants to transplanting events between latitudes.

**Supplementary Information:**

The online version contains supplementary material available at 10.1186/s12870-021-03123-1.

## Background

Mangroves are woody plants growing in the interface between land and sea in tropical and sub-tropical areas [1]. They have morphological and physiological characteristics to adapt to stressful conditions such as high salinity, strong winds, and tides, high temperatures, anaerobic soils, etc. [1]. Mangrove forests play important roles in protecting and stabilizing coastlines, producing commercial forest products, and supporting coastal fisheries, having enormous ecological values [2]. As one of the most productive ecosystems, mangrove forests are outstanding in producing organic carbon and contribute significantly to the global carbon cycle [3]. However, the unprecedented loss of mangrove ecosystems all over the world has aroused many concerns from the scientific community [4]. In the 1980s and 1990s, it was suggested 35% of the world's mangrove forests had been lost because of deforestation [5]. Hamilton and Casey's estimation showed annual mangrove forest loss average 0.26–0.66% globally between 2000 and 2012 [6]. According to a survey by the State Forestry Administration of China, only 44% of original mangrove forests of the 1950s in China remained in 2002 [7]. Although 12% of China's mangrove forests have been recovered from 2000 to 2015 [8], mangrove protection and restoration still face many challenges [9, 10].

*Kandelia obovata* is one of the most widely distributed and cold-resistance mangrove species in China [11]. Previous studies observed vegetative morphology variations of *K. obovata* with different latitudes [12]; and a dwarf phenotype was found at high latitude [13]. The force of natural selection, resulting in interactions of genotype and environment for Darwinian fitness, should cause the local population to evolve traits that give advantages under its local environmental conditions [14]. In the absence of other forces and constraints, resident genotypes in each local population would show on average a higher relative fitness in their local habitat than genotypes originating from other habitats. This pattern, including the process leading to it, is local adaptation [14]. Using reciprocal transplants experiments, the ‘gold standard’ for detecting local adaptation, reciprocal adaptation has been found in 45% of studies in plants [15]. In research on *Arabidopsis*, transplanted individuals have a lower survival rate and lower fecundity in addition to changes in flowering time [16].

Plants can also acclimatize and respond rapidly to environmental changes. Phenotypic changes involve modifications in gene expression. DNA methylation, which is a process that adding a methyl group to a cytosine base, is an epigenetic modification that constitutes an additional level of regulation of gene expression without affecting the underlying DNA sequences [17]. There is emerging evidence that DNA methylation plays an important role in regulating plant development and morphology [18–22]. Epigenetic regulation of rapid responses to environmental fluctuation and phenotypic variation has also been observed in many organisms, such as reef corals [23] and alligator weed [24]. Early studies on responses to abiotic stresses showed stress-induced DNA methylation and/or demethylation patterns either genome-wide or at specific loci [25]. DNA methylation changes may also be associated with transcriptional regulation [26–29]. These results all indicate that epigenetic modifications are responsible for environmental sensitivity and flexibility of plants.

As far as we know, there has been no experiment performed to study the association of the epigenetic regulations and local adaptation in mangrove plants yet. Here, we sampled seedings of *K. obovata* from three populations along latitude gradients in China and performed seven-year reciprocal transplant experiments combined with common garden experiments in the field. Phenotypes, including height, crown width, and basal stem diameter, were measured. Taking advantage of the whole genome re-sequencing (WGRS), the transcriptome sequencing (RNA-seq), and the whole-genome bisulfite sequencing (WGBS), our research aims to explore whether there is adaptive differentiation in the three *K. obovata* populations and to study how *K. obovata* responded to the environmental changes induced by transplantation.

## Results

### Morphology differences among *K. obovata* from different populations and their phenotypic plasticity

**Fig. 1:**
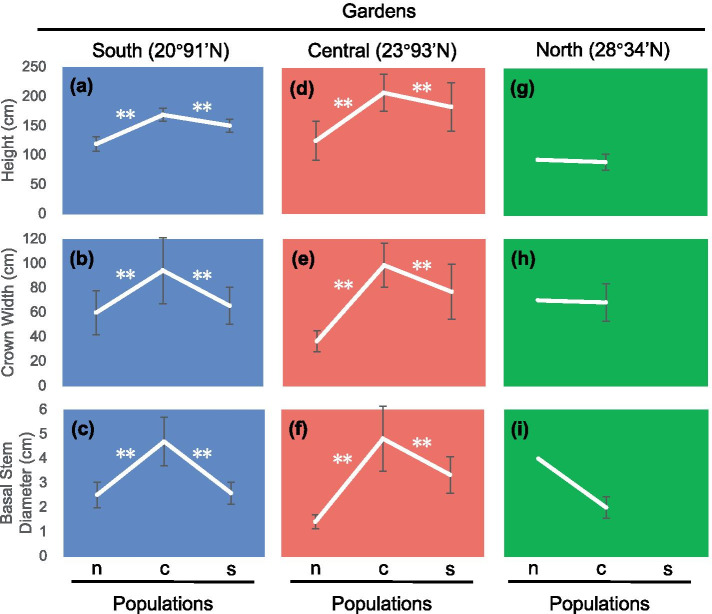
Phenotypic differences among populations. Means for height ((**a**), (**d**), (**g**)), crown width ((**b**), (**e**), (**h**)) and basal stem diameter ((**c**), (**f**), (**i**)) of plants that collected from north, central and south populations while be planted in South garden (bule box), Central garden (red box) and North garden (green box). Bars indicate standard errors. Significant differences are shown * (*P* < 0.05) or ** (*P* < 0.01)

**Fig. 2:**
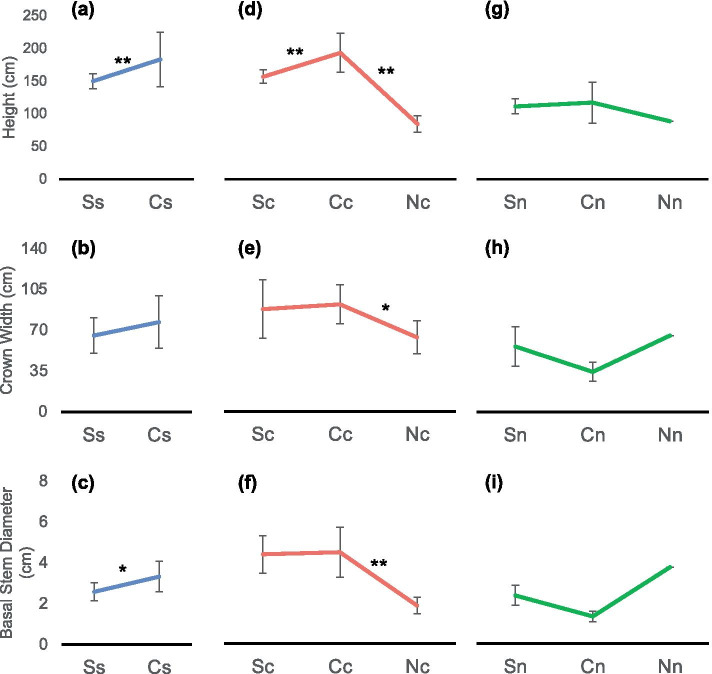
Phenotypic changes after transplantation. Transplant-induced phenotypic changes of individuals from the south population (blue line), the central population (red line), and the north population (green line). Bars indicate standard errors. Significant differences are shown * (*P* < 0.05) or ** (*P* < 0.01)

We conducted seven-year-long common garden experiments and reciprocal transplant experiments in our study. In South and Central gardens, significant morphological differences among individuals that from different populations were observed (Fig. 1a-f): the plants from the central populations grew faster than plants from the other two populations, showing obvious advantages in height (Fig. 1a, d), crown width (Fig. 1b, e) and basal stem diameter (Fig. 1c, f). While in the North garden, the plants from the south population did not survive and plants from the central population showed significant growth inhibition (Fig. 1i). In the Central garden, the local individuals showed significant dominance of height, crown width, and basal stem diameter (Fig. 1d-f). Besides, the central population displayed growth inhibition after transplantation (Fig. 2d-f), also indicating local adaptation. While in the South garden, plants from the south population did not show any local advantages (Fig. 1a-c). Furthermore, the growth rate of plants from the south populations increased after transplanted into the Central garden (Fig. 2a-c).

For *K. obovata* from each population mentioned above, changes in morphology were observed after being transplanted to other latitudes (Fig. 2). Populations differed in their phenotypic plasticity (Table 1): central population in height, north population in crown width, as well as basal stem diameter, had the greatest phenotypic plasticity. The greatest mean phenotypic plasticity was found in the plants from the central population.

**Table 1:** Index of phenotypic plasticity

Population	Height	Crown Width	Stem Diameter	Average
south	44.44%	14.95%	22.33%	27.24%
central	56.29%	30.80%	58.48%	48.52%
north	24.34%	47.62%	64.44%	45.47%

### Genetic differentiation of *K. obovata* populations

**Fig. 3:**
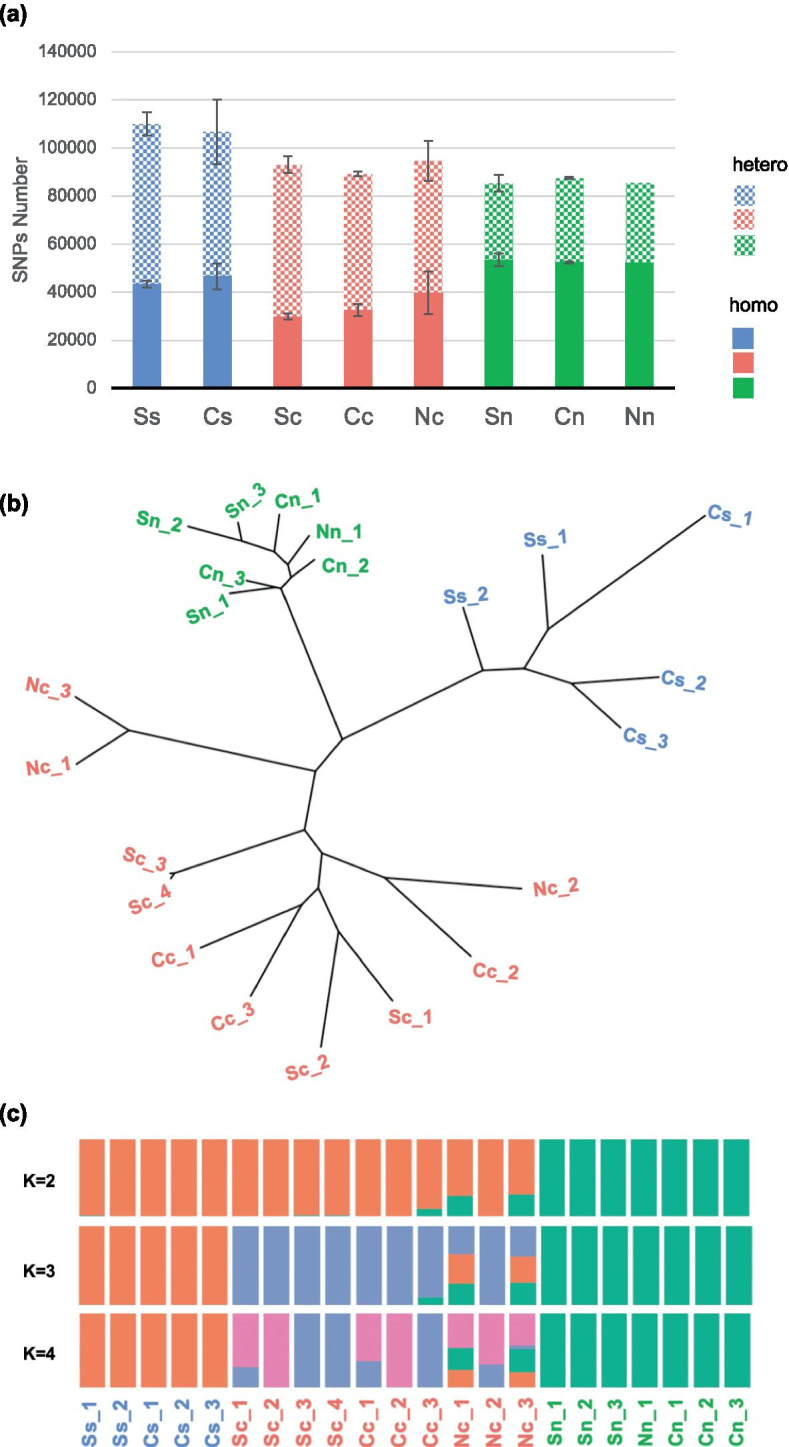
Genetic variations of the twenty-two *K. obovata* individuals. (**a**) Bar chart showing differences of SNP numbers among and within south (blue), central (red), and north (green) population. Mosaic: heterozygous SNP, solid: homozygous SNP. Bars indicate standard errors. (**b**) Phylogenetic tree of twenty-two individuals from the south (blue), central (red), and north (green) populations. (**c**) Population structure inferred with admixture from the same data set for a different number of ancestral clusters (K = 2 to 4). The best partition was obtained for K = 2 ancestral clusters. Each of the twenty-two individuals was represented by a vertical bar

Observing morphology differentiation among *K. obovata* populations from three latitudes, we further explored its genetic basis by taking advantage of whole-genome re-sequencing of *K. obovata* from those three populations. Single nucleotide polymorphism sites (SNPs) were summarized after mapping the trimmed high-quality reads to the reference genome (Table S1). For a single individual, 84,264 to 112,414 SNPs were identified from at least 61 million cleaned reads. The most abundant SNPs were identified in south-population samples, while the minimum numbers of SNPs were found in north-population samples. On average over 60% of SNPs identified in the north-population samples were homozygous. There were only ~ 36% and ~ 42% homozygous SNPs in central- population ones and south-population ones, separately (Fig. 3a).

In terms of the numbers of SNPs only, bigger differences among populations than planting environments were observed (Fig. 3a). Therefore, we expected there is genetic differentiations among *K. obovata* populations from different latitudes. The maximum-likelihood genome genealogy of twenty-two individuals confirmed our hypothesis (Fig. 3b). Phylogenetic analysis clustered *K. obovata* individuals into three classes and the distribution correspond to their populations. Additionally, individuals of the north population clustered more closely with each other, indicating that there is less diversity among the north population.

We further performed population structure analysis using ADMIXTURE, which estimates individual ancestry and admixture proportions assuming K ancestral populations (Fig. 3c). At K = 2, the north population emerged as a single cluster. The ADMIXTURE analysis at K = 3 grouped populations by their provenances. With increasing K values, a substantial proportion of other ancestry was observed in the central population, reflecting historical gene flow among these populations.

Using phylogenetic and ADMIXTURE analyses, we observed a clear genetic differentiation among south, central and north populations, which may lead to previously observed morphology differentiation. We also noticed that genetic diversity is highest within the central population and lowest within the north population.

### Transplant-induced inhibition of lignin biosynthesis and reduction of biomass

**Fig. 4:**
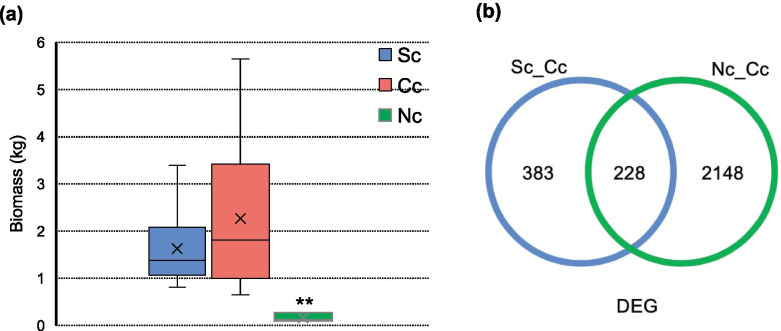
Changes of biomass and gene expression induced by transplantation. (**a**) Biomass of central population individuals, including the southward-transplant (Sc, blue), the local (Cc, red), the northward-transplant (Nc, green). (**b**) DEG numbers of Sc (blue circle) and Nc (green circle) relative to Cc

Transplant-induced reduction of biomass was observed in both northward and southward transplantation (Fig. 4a). The average biomass of a single individual decreased from 2.26 kg to 0.16 kg after the northward transplantation. Transcriptome sequencing was carried out on leaves of those nine central-population samples, including three that transplanted to South garden (Sc), three to North garden (Nc), and three in Central garden (Cc). Principal component analysis (PCA) of expression data showed clear separation corresponding to the locations where the plants were planted (Fig. S2), indicating that transcriptomic profiles were affected by transplanting. Relative to Cc, 611 and 2,376 differentially expressed genes (DEGs) were revealed in Sc and Nc separately (Fig. 4b). Genes expressed unconventionally after northward transplanted, it is probably because of greater environmental differences between Central garden and North garden. Circadian rhythm, flavonoid, and phenylpropanoid biosynthesis-related genes prevailed at the top of the KEGG pathway enrichment of DEGs (Fig. S3-S4). Phenylalanine ammonia-lyase (*PAL*), which catalyzes the first step in the phenylpropanoid pathway, was down-regulated significantly both in Sc and Nc (Table S2, Fig. S5). Lignin is synthesized from phenylpropanoid compounds. Moreover, 4-coumarate:CoA ligase (*4CL*) and ferulate-5-hydroxylase (*F5H*), as the important lignin biosynthesis enzymes, were also significantly down-regulated (Table S2). Consistent with previous studies, our transcriptome analysis revealed lignin biosynthesis inhibition and biomass reduction might cause by reducing the expression of *PAL*, *4CL*, and *F5H*.

### DNA methylome of *K. obovata* kept robust after transplanting and local modification involved transcription and translation regulation

**Fig. 5:**
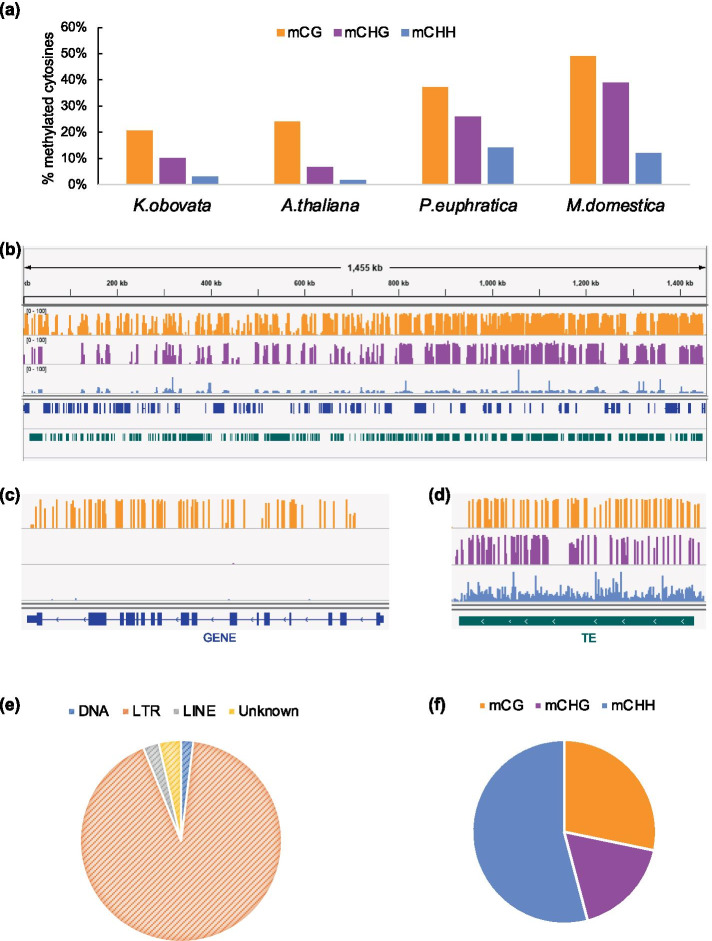
DNA methylation level and distribution in *K. obovata*. (**a**) Average methylation level in CG (orange), CHG (purple), CHH (blue) sequence context of *K. obovata*, *A. thaliana*, *P. euphratica*, *M. domestica*. (**b**) Integrative Genomics Viewer depicts methylation level and distribution of mC in Contig64. (**c**) Integrative Genomics Viewer depicts methylation level and distribution of mC on a gene. (**d**) Integrative Genomics Viewer depicts methylation level and distribution of mC on a transposable element. (**e**) The pie chart showing a preference of mC for different types of transposons. (**f**) The pie chart showing a preference of mC for different sequence contexts

We sequenced about 700 million pair-end 150-bp reads(~ 112 Gb)of bisulfite-converted DNA (conversion rate > 99.8%), which yielded an average depth of ~ 30 × per strand. At the whole genome level, we found DNA methylation levels of CG, CHG, and CHH (where H is adenine, thymine, or cytosine) were 20.70%, 10.17%, and 3.07%, respectively. DNA methylation sites are not evenly spread and methylated cytosines tend to locate in the transposon enriched regions. In the gene enriched regions with less transposon, only methylated cytosines in CG are observed (Fig. 5b). We further investigated the methylation level in genic regions and transposable element (TE) regions. TEs are extensively methylated as expected (Fig. 5d), whereas only cytosines in the CG are highly methylated, while CHG and CHH methylation are almost depleted in the gene body (Fig. 5c).

We defined the sites that have a minimum of five methylated cytosine reads as methylation site (mC site). In total, near two million mC sites were detected in the whole genome, which covered 2.52% of cytosines in the *K. obovata* genome. In these mC sites, ~ 9% of them were located in genes, but almost 88% were found in transposons especially in the LTR retrotransposons (Table S2 and Fig. 5e). As for context bias, more than half of mC sites were detected in the CHH contexts, with about 29% of CG and ~ 17% CHG (Fig. 5f).

**Fig. 6:**
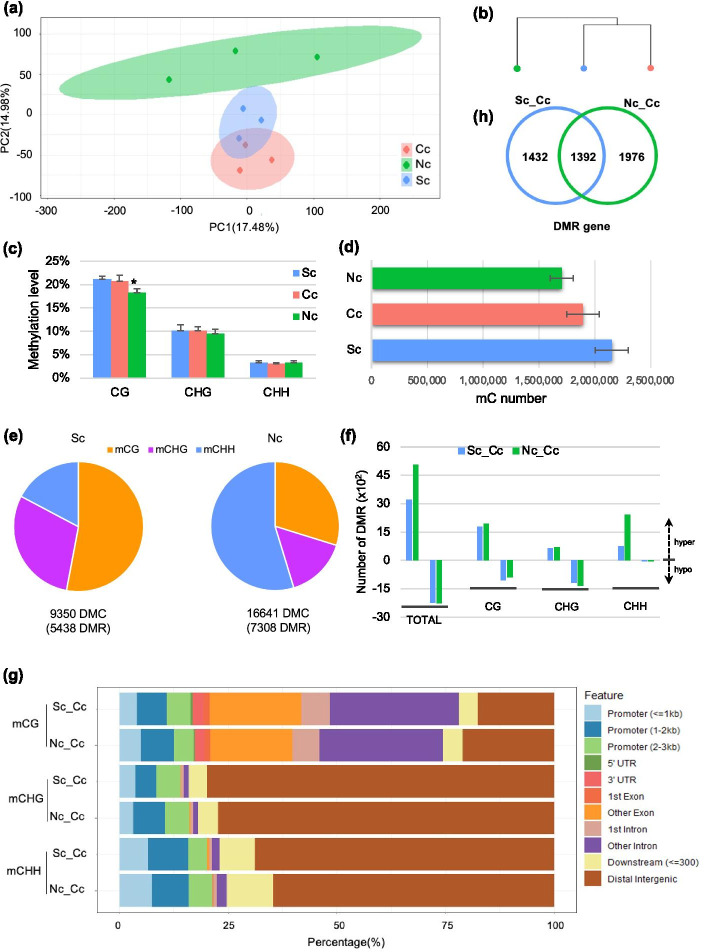
Transplant-induced changes of DNA methylation profiles. (**a**) PCA of DNA methylation profiles of nine individuals from the central population, including the southward-transplant (Sc, blue), the local (Cc, red), the northward-transplant (Nc, green). (**b**) Correlation of the methylation profiles of the transplanted and un-transplanted. (**c**) Bar chart showing average methylation level differences induced by transplantation. (**d**) Methylated cytosine number of the transplanted and un-transplanted. (**e**) The total number and sequence context breakdown of DMCs induced by transplantation. (**f**) The number and direction of methylation changes at CG, CHG, CHH. (**g**) Stacked bar graph showing the percentage of the DMRs overlapping with various annotated genic regions, including promoter, UTRs, exon, intron, and distal intergenic region, relative to the total numbers of DMRs associated with these genic features in each sequence context at Nc or Sc. (**h**) DMR-gene number of Sc (blue circle) and Nc (green circle)

Wondering how DNA methylation response to transplantation, we compared methylomes of the nine central-population samples (Sc, Nc, and Cc). Principal component analysis (PCA) results based on methylation profiles showed a clear separation according to the latitudes (Fig. 6a). The methylation profile of Cc had a higher similarity with Sc than Nc (Fig. 6b), suggesting that the methylation profile might be determined by the planted environment. The genome-wide methylation levels at mCGs, mCHGs, mCHHs were similar across samples, except for a drop in CG context after northward transplanting (Fig. 6c), indicating that a complete and pancellular reprogramming of DNA methylation did not happen after transplanting. There is a negative correlation between latitude and the number of mC sites, where fewer mC sites were detected in samples planted at higher latitude (Fig. 6d).

To examine the extent of dynamic regulations of DNA methylation that occurred locally in response to the planted environment, differentially methylated cytosines (DMCs) and differentially methylated regions (DMRs) were further identified. We found northward-transplanting (Nc) induced more DMC than southward-transplanting (Sc) (Fig. 6e). DMCs in Nc were largely composed of cytosines in the CHH sequence context, whereas DMCs in the CG sequence context were the most abundant in the Sc samples (Fig. 6e). We observed hyper- and hypomethylation of DMRs in all sequence contexts (Fig. 6f). In addition, we found that DMRs in CHG and CHH sequence contexts were strongly enriched in intergenic regions (Fig. 6g), whereas DMRs in the CG sequence context occurred predominantly in the gene bodies (exons and introns).

After annotating transplant-induced DMRs (Fig. 6e) to their genetic location, we obtained 3,368 and 2,824 DMR genes in Nc and Sc, separately (Fig. 6h). To explore the biological roles of those differentially methylated genes, KEGG enrichment analyses were performed. For DMR-genes, the pathways ‘spliceosome’ and ‘RNA transport’ were significantly enriched both in Nc and Sc (Fig. S6-S7), suggesting that transplant-induced local modification of DNA methylation in *K. obovata* involved in the regulation of transcription and translation.

### Correlation between expression changes and DNA methylation modification in transplanted *K. obovata*.

**Fig. 7:**
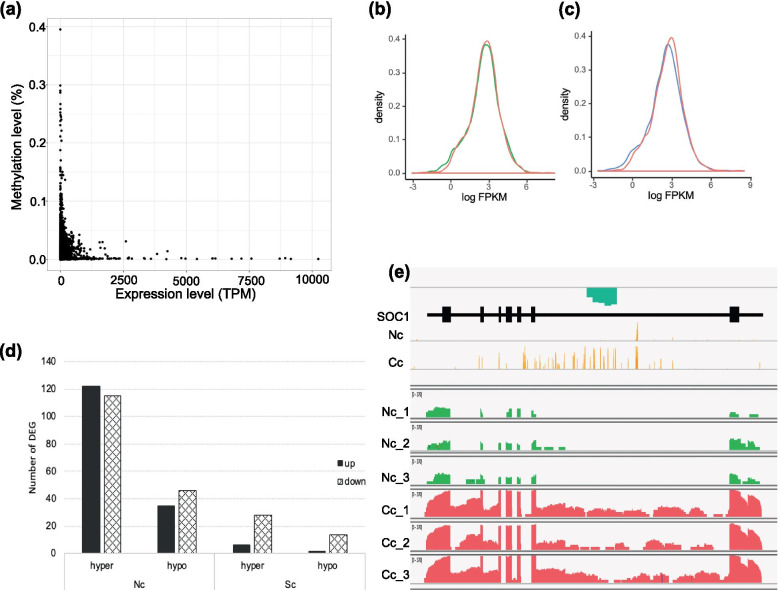
Correlation of DNA methylation and gene expression. (**a**) The DNA methylation level and expression level of all the genes in *K. obovata*. (**b**) Density curve of the expression level of DMR-genes in Nc (green line), relative to Cc (red line). (**c**) Density curve of the expression level of DMR-genes in Sc (blue line), relative to Cc (red line). (**d**) Correlation of DEGs and DNA methylation modification. (**e**) Visualization of expression level and methylation level of SOC1 in Cc and Nc. Orange: methylation level in CG context. Blue box: hypo-methylation regions. Green peak: RNA-seq reads coverage in Nc. Red peak: RNA-seq reads coverage in Cc

In all 23,683 genes in the *K. obovata* genome, the highly methylated genes were rarely expressed; and the genes with high expression were usually with very low methylation level (Fig. 7a). The transcript profile of transplant-induced DMR genes was changed (Fig. 7b, c). Moreover, we noticed that more than 80% of DEGs were with extremely low methylation levels (Fig. S8-S9), indicating the role of DNA methylation in maintaining the stability of gene expression. Among the 2,759 transplant-induced DEGs, there were only 347 genes where DMRs were detected. No strong overlap of DMRs and DEGs were observed in this study (Fig. 7d), suggesting that DNA methylation may precede transcriptional changes in a rather complicated way.

Among the 347 differentially expressed as well as differentially methylated genes, 27 of them were presumed as transcription factors (Table. S3). It is noteworthy that transcription factors of the MADS-box family, which function in the formation of flowers and the control of flowering time, are most abundant in the list. All of these five MADS-box genes were significantly down-regulated after northward-transplantation. Moreover, demethylation in the CG context was observed in the intron of these five genes (Table S4, Fig. 7e).

## Discussion

*K. obovata* is one of the most widely distributed mangrove species in China. The center of *K. obovata’s* geographic distribution in China is located near Yunxiao. Plants with the south (Leizhou) and central (Yunxiao) population grew faster in the Central garden (Yunxiao) than in the South garden (Leizhou) (Fig. 2a-f). For the north population, the individuals planted in North garden have higher biomass than those in Central garden (Fig. 2g-i). In addition, the north population showed advantages in the north garden, which suggests their better abilities in cold tolerance of *K. obovata* from the north population. Genetic differentiation was found among south, central and north populations (Fig. 3), which might be responsible for the different preferences of these three populations for different latitudes. Moreover, relatively low genetic diversity of the north population was observed. According to the World Conservation Union, genetic diversity is one of the biodiversity that deserving conservation [30]. In addition, mangrove species with low genetic diversity were more vulnerable to destruction [31]. Effective protection needs to be carried on to the north population, which also had a small population size and remarkable cold resistance.

The process of *K. obovata* afforestation, where individuals were transplanted to a novel environment, are essentially human-controlled migration events. Phenotypic plasticity offers plants a mechanism to tolerate wide environmental variations [32]. Genetic variations can contribute to a higher survival rate [32]. There were many pieces of research showed that genetic diversity and phenotypic plasticity helped plants survive in the novel environment [32–34]. High genetic diversity and phenotypic plasticity (Fig. 2d-f, Fig. 3) make the seedings from the central (Yunxiao) population the best choice for plantation. Nevertheless, the north (Ningde) population, with strong cold tolerance, might be more suitable to be planted in the higher latitudes.

After transplanted to other latitudes, the individuals from the Yunxiao population declined in biomass (Fig. 4a). A research of *Populus* observed negative correlation (*r* =  − 0.48; *P* value < 0.0001) between biomass and lignin content [35]. A mutant of aspen (*Populus tremuloides*) with 45% less lignin, showing down-regulated of 4-coumarate:coenzyme A ligase (*4CL*) genes. Lignin is produced by the plant phenylpropanoid pathway. In our case, phenylalanine ammonia-lyase (*PAL*), which catalyzes the first step of the phenylpropanoid pathway [36], was significantly decreased after being planted outside of its native habitats. Besides, the expressions of monolignol biosynthetic enzymes, *4CL* and *F5H*, were both reduced significantly. Inhibition of *PAL*, *4CL, or F5H* leads to reduced lignin content in plants [37–40]; and *pal* knockout mutants were stunted and sterile [36]. In plants, lignin was a component of the secondary cell wall [41]. A substantial fraction of weight of trees is made up by lignin, as the wood of trees is composed mainly of secondary cell walls of vascular tissue and accompanying fibers [42]. We speculate that transplant-induced biomass reduction was correlated to inhibition of the expression of lignin biosynthesis genes, like *PAL*, *4CL*, and *F5H*.

DNA methylation is a conserved epigenetic modification that plays an important role in gene regulation [25]. DNA methylation patterns could broadly reflect the evolutionary and life histories of plant species [43]. There are extensive variations throughout angiosperms in DNA methylation [43]. *K. obovata* has a relatively lower methylation level (21%, 10%, 3%) compared with these in other woody plants, eg. popular (37%, 26%, 14%) [44] or apple (49%, 39%, 12%) [45], but the level is similar as observed in *Arabidopsis* (24%, 7%, 2%) [46]. Previous research has shown that genomic methylation is related to genome size, that is angiosperms with larger genome size methylate a higher proportion of their genomes [47, 48]. Both the genome size of apple (651 Mb) [45] and the populus (485 Mb) [49] are larger than the genome of *K. obovata* (278 Mb), which might explain the relatively low methylation level of *K. obovata* (Fig. 5a). Accumulating evidence has demonstrated the environment-respond altering of plant DNA methylation at individual loci or across the entire genome, but DNA methylation modification only accounted for limited gene expression variations [50–52]. The associations between DNA methylation and gene expression are complex and context-dependent [53]. The role of DNA methylation in plants depends upon the underlying sequence and the location in the genome, instead of being simply on or off [54]. We observed CG demethylation in those transplant-induced down-regulated MADS-box genes, and the demethylation all occurred in the introns (Table S4). DNA methylation is found to be essential in the process of vernalization and flowering [55–58]. MADS-box genes, like *FUL*, *SVP*, and *SOC1*, are reported to involve in regulating flowering time [59–64]. In the lifetime of a plant, flowering is a critical developmental stage that is very vulnerable to environmental stresses. Adjusting flowering time is an evolutionary strategy adopted by plants [65]. In addition, *SOC1* has been shown to account for the cross-talk between cold response and flowering. The *soc1* mutants showed increased cold tolerance and delayed flowering time [66]. The seasonal periods conducive to growth and reproduction are geographically variable. Generally, flowering time follows a latitudinal gradient. For example, *Arabidopsis thaliana* tended to flower later in the North and earlier in the South [67]. When transplanted to other latitudes, DNA methylation modification may help *K. obovata* adapt to the new environment by adjusting flowering time. We did notice that *K. obovata* plants flowered earlier in the southern common gardens in some cases. However, we usually visit the gardens on a monthly basis, the time span is too long to accurately determine the actual flowering times of plants in three gardens. Further research and experimental verification are still needed to explore the specific regulatory mechanisms.

## Conclusions

Based on our study, we observed morphology differences and genetic differentiation among *K. obovata* from different populations and call for more attention on the protection of *K. obovata* especially from the north (Ningde) population, which had low genetic diversity and small population size but outstanding cold resistance. In addition, this study could offer a scientific basis to choose the proper seeding for plantation to efficiently restore. By exploring phenotypic, transcript, and epigenetic responses to transplanting events, our work shed light on revealing the molecular mechanism of *K. obovata* responding to environmental changes.

## Methods

### Plant growth and phenotypic measurement

The study was conducted on open mudflats on the southern coast of China. Three planting sites were chosen along a latitudinal gradient, Leizhou (20°91′N), Yunxiao (23°93′N), and Yueqing (28°34′N). Hypocotyls of *K. obovata* were collected from natural mangrove forests in Leizhou (20°91′N), Yunxiao (23°93′N) and Ningde (27°28′N), the northern boundary of naturally grown mangroves in China, in Spring of 2011 and then were reciprocally transplanted in the common gardens located in intertidal mudflats in chosen planting sites respectively (Fig. S1). The samples in our study were obtained from the wild and no permission was necessary. Prof. Yihui Zhang undertook the formal identification of the samples and provide details of any voucher specimens deposited. Our experiments on plants comply with institutional, national, or international guidelines. Our field studies were conducted in accordance with local legislation. All necessary permits for planting were obtained from Xiamen University, China.

From late March to early April of 2018, in each site, we selected individuals randomly from three different latitudes, with three biological duplications each. Due to mislabeling and deaths from natural disasters, twenty-two individuals were obtained (Table S1), including three Cs (individuals collected from south population and planted in Central garden), two Ss (individuals collected from south population and planted in South garden), four Sc (individuals collected from central population and planted in South garden), three Cc (individuals collected from central population and planted in Central garden), three Nc (individuals collected from central population and planted in North garden), three Sn (individuals collected from north population and planted in South garden), three Cn (individuals collected from north population and planted in Central garden), one Nn (individuals collected from north population and planted in North garden). In each garden, fresh leaves were collected and quickly frozen in dry ice between 9:00 and 11:00. Phenotypes were measured on the spot, including plant height, basal stem diameter (30 cm above the soil surface), and crown width (the lengths of the major and the minor axes of the canopy). To compare the extent of phenotypic changes induced by transplantation, an index of phenotypic plasticity was calculated for each population and variables as the difference between the maximum and the minimum mean values among the three planting environments divided by the maximum mean value.

To study the mechanism of phenotypic changes induced by transplantation, we focused on the nine central-population individuals (yellow ones in Table S1) and the aboveground biomass (*w*_T_) of each individual was predicted based on tree height (H) and stem diameter (D) [68]:

$${w}_{T} = 0.04117({D}^{2}H)$$

### Library preparation and sequencing

For each sample, DNAs and RNAs were obtained from the same piece of leaf. Total RNAs were extracted by RNAprep Pure Plant Plus Kit (TIANGEN, Beijing, China, DP441). Total genomic DNAs were extracted through DNAquick Plant System (TIANGEN, Beijing, China, DP321).

For RNA-seq, the libraries were constructed using NEBNext® UltraTM RNA Library Prep Kit for Illumina® (NEB, USA) following the manufacturer's recommendations, and index codes were added to attribute sequences to each sample. Briefly, mRNA was purified from total RNA using poly-T oligo-attached magnetic beads. Fragmentation was carried out using divalent cations under elevated temperature in NEBNext First Strand Synthesis Reaction Buffer (5X). First-strand cDNA was synthesized using random hexamer primer and M-MuLV Reverse Transcriptase (RNase H). Second strand cDNA synthesis was subsequently performed using DNA Polymerase I and RNase H. Remaining overhangs were converted into blunt ends via exonuclease/polymerase activities. After adenylation of 3’ends of DNA fragments, NEBNext Adaptor with hairpin loop structure was ligated to prepare for hybridization. 250 ~ 300 bp fragments were purified with AMPure XP system (Beckman Coulter, Beverly, USA). Then 3 μl USER Enzyme (NEB, USA) was used with size-selected, adaptor-ligated cDNA at 37 °C for 15 min followed by 5 min at 95 °C before PCR. Then PCR was performed with Phusion High-Fidelity DNA polymerase, Universal PCR primers, and Index (X) Primer. At last, PCR products were purified (AMPure XP system), and library quality was assessed on the Agilent Bioanalyzer 2100 system.

DNAs of each sample was divided into two parts and used for whole-genome re-sequencing (WGRS) and whole-genome bisulfite sequencing (WGBS) separately. WGRS libraries were obtained using Truseq Nano DNA HT Sample Preparation Kit (Illumina USA) following instructions with 1.5 μg DNA as input. Briefly, DNA was fragmented to 350 bp by sonication, then DNA fragments were end-polished, A-tailed, and ligated with the full-length adapter for Illumina sequencing with further PCR amplification. At last, PCR products were purified (AMPure XP system).

As for WGBS libraries, a similar strategy with WGRS’s was followed. But unlike WGRS, DNA was fragmented to 200–300 bp and barcodes were cytosine-methylated. DNA fragments were treated twice with bisulfite using EZ DNA Methylation-GoldTM Kit (Zymo Research), and then these bisulfite-converted DNA fragments were amplified using KAPA HiFi HotStart Uracil + ReadyMix (2X).

All these libraries were analyzed for size distribution by Agilent2100 Bioanalyzer and quantified using real-time PCR. Subsequently, libraries were sequenced in Novogene Company (Beijing, China) using Illumina Hiseq X platform and 150 bp paired-end reads were generated (Raw reads were placed in GEO under submission number SUB7839708).

### Analyses of sequencing data

A quality control process was applied to reads without sequencing adapters and primers sequences by Trimmomatic [69]. Low-quality nucleotides (Q < 20) were discarded from both ends of reads and reads shorter than 50 bp were removed (SLIDINGWINDOW 5:20, LEADING 5, MINLEN 50).

For each individual, trimmed reads were mapped to the *K. obovata* reference genome with Burrows-Wheeler Aligner (BWA v.0.7.17) [70], using default parameters. Duplicated reads were marked and variant calling was performed with GATK (v.4.0.10.1) [71]. Variants were then filtered using GATK, and the qualified SNPs were used to construct the phylogenetic relationship using maximum likelihood by SNPhylo [72]. Admixture in individuals was estimated using the qualified SNPs with ADMIXTURE [73].

Trimmed RNA-seq reads were mapped to the *K. obovata* reference genome using HISAT2 [74]. Following mapping, RNA-Seq alignments were assembled into potential transcripts using StringTie [75]. Differentially expressed genes were determined using DEseq2 [76] implemented in R.

For trimmed WGBS reads, mapping and methylation calling were performed by Bismark (v.0.20.0) [77]. For each sample, reads were aligned to a sample-specific modified pseudo reference in which homozygous SNPs were inserted into the *K. obovata* reference genome. Differentially Methylated Cytosines (DMCs) and differentially Methylated Regions (DMRs) were identified using methylKit (v.1.10.0) [78]. When identifying DMRs, the genome was tiled with windows 100 bp length and 100 bp step-size, and the methylation information on those tiles was summarized. Also, for CG, CHG, and CHH context sequences, DMC/DMR candidates with the ratio of less than 40%, 20%, and 10% differences between maximum and minimum methylation levels were discarded, respectively.

### qRT-PCR validation of gene expression

The expression levels of PAL, 4CL, and F5H were confirmed by qRT-PCR. First-strand cDNA was synthesized using SMARTScribe reverse transcriptase (Clontech) and then used to perform qRT-PCR using SYBR Premix on Bio-Rad CFX 96 (Bio-Rad, Inc.), according to the manufacturer's instructions. The relative quantification from three biological replications was normalized to the reference gene, 18S rRNA (accession no. AY289625), and calculated by the 2 − ΔΔCt method. All primer sequences are shown in Table S5.

## Supplementary Information

**Additional file 1.**

## Data Availability

Sequencing data generated for this study have been deposited in NCBI and are available at https://dataview.ncbi.nlm.nih.gov/object/PRJNA648812?reviewer=90naarf2cn2ua81g2r0aec5m3a.

## Abbreviations

K. obovata: Kandelia obovata
WGRS: Whole genome re-sequencing
WGBS: Whole genome bisulfite sequencing
SNP: Single nucleotide polymorphism
DEG: Differentially expressed gene
KEGG: Kyoto encyclopedia of genes and genomes
PAL: Phenylalanine ammonia-lyase
4CL: 4-Coumarate:CoA ligase
F5H: Ferulate-5-hydroxylase
TE: Transposable element
PCA: Principal component analysis
DMC: Differentially methylated cytosine
DMR: Differentially methylated region

## References

[1] Tropical Mangrove Ecosystems. 1st edition. American Geophysical Union (AGU); 1992. doi:10.1029/CE041.

[2] Saenger P. Mangrove Ecology, Silviculture and Conservation. Springer Science & Business Media; 2002.

[3] Kathiresan K, Bingham BL. Biology of mangroves and mangrove Ecosystems. In: Advances in Marine Biology. Elsevier; 2001. p. 81–251. doi:10.1016/S0065-2881(01)40003-4.

[4] Bosire JO, Dahdouh-Guebas F, Walton M, Crona BI, Lewis RR, Field C (2008). Functionality of restored mangroves: A review. Aquat Bot.

[5] Valiela I, Bowen JL, York JK (2001). Mangrove Forests: One of the World’s Threatened Major Tropical Environments: At least 35% of the area of mangrove forests has been lost in the past two decades, losses that exceed those for tropical rain forests and coral reefs, two other well-known threatened environments. Bioscience.

[6] Hamilton SE, Casey D (2016). Creation of a high spatio-temporal resolution global database of continuous mangrove forest cover for the 21st century (CGMFC-21). Glob Ecol Biogeogr.

[7] Jia M, Wang Z, Li L, Song K, Ren C, Liu B (2014). Mapping China’s mangroves based on an object-oriented classification of Landsat imagery. Wetlands.

[8] Jia M, Wang Z, Zhang Y, Mao D, Wang C (2018). Monitoring loss and recovery of mangrove forests during 42 years: The achievements of mangrove conservation in China. Int J Appl Earth Obs Geoinf.

[9] Ellison AM (2000). Mangrove Restoration: Do We Know Enough?. Restor Ecol.

[10] Alongi DM (2002). Present state and future of the world’s mangrove forests. Environ Conserv.

[11] Fei J, Wang Y, Cheng H, Su Y, Zhong Y, Zheng L (2021). Cloning and characterization of KoOsmotin from mangrove plant Kandelia obovata under cold stress. BMC Plant Biol.

[12] Chen G, Dai C, Li Y, Xu J. Comparison of morphological characteristics of mangrove plants in coastal areas of Fujian province. Journal of Anhui Agricultural Sciences. 2016;44:178–182+253.

[13] Shi X, Pan L, Chen Q, Huang L, Wang W (2016). Dwarf reasons of mangrove plant *Kandelia obovata* in Shancheng bay. Wetland Science.

[14] Kawecki TJ, Ebert D (2004). Conceptual issues in local adaptation. Ecol Lett.

[15] Leimu R, Fischer M. A meta-analysis of local adaptation in plants. PLoS ONE. 2008;3:e4010. 10.1371/journal.pone.0004010 PMC2602971 19104660

[16] Ågren J, Schemske DW (2012). Reciprocal transplants demonstrate strong adaptive differentiation of the model organism Arabidopsis thaliana in its native range. New Phytol.

[17] Suzuki MM, Bird A (2008). DNA methylation landscapes: provocative insights from epigenomics. Nat Rev Genet.

[18] Ronemus MJ, Galbiati M, Ticknor C, Chen J, Dellaporta SL (1996). Demethylation-Induced Developmental Pleiotropy in Arabidopsis. Science.

[19] Burn JE, Smyth DR, Peacock WJ, Dennis ES (1993). Genes conferring late flowering inArabidopsis thaliana. Genetica.

[20] Finnegan EJ, Peacock WJ, Dennis ES (1996). Reduced DNA methylation in Arabidopsis thaliana results in abnormal plant development. Proc Natl Acad Sci U S A.

[21] Kooke R, Johannes F, Wardenaar R, Becker F, Etcheverry M, Colot V (2015). Epigenetic Basis of Morphological Variation and Phenotypic Plasticity in Arabidopsis thaliana. Plant Cell.

[22] Zhong S, Fei Z, Chen Y-R, Zheng Y, Huang M, Vrebalov J (2013). Single-base resolution methylomes of tomato fruit development reveal epigenome modifications associated with ripening. Nat Biotechnol.

[23] Dimond JL, Roberts SB (2016). Germline DNA methylation in reef corals: patterns and potential roles in response to environmental change. Mol Ecol.

[24] Gao L, Geng Y, Li B, Chen J, Yang J (2010). Genome-wide DNA methylation alterations of Alternanthera philoxeroides in natural and manipulated habitats: implications for epigenetic regulation of rapid responses to environmental fluctuation and phenotypic variation. Plant, Cell Environ.

[25] Zhang H, Lang Z, Zhu J-K (2018). Dynamics and function of DNA methylation in plants. Nat Rev Mol Cell Biol.

[26] Xu R, Wang Y, Zheng H, Lu W, Wu C, Huang J (2015). Salt-induced transcription factor MYB74 is regulated by the RNA-directed DNA methylation pathway in Arabidopsis. J Exp Bot.

[27] Zhang B, Tieman DM, Jiao C, Xu Y, Chen K, Fei Z (2016). Chilling-induced tomato flavor loss is associated with altered volatile synthesis and transient changes in DNA methylation. PNAS.

[28] Khan AR, Enjalbert J, Marsollier A-C, Rousselet A, Goldringer I, Vitte C (2013). Vernalization treatment induces site-specific DNA hypermethylation at the VERNALIZATION-A1 (VRN-A1) locus in hexaploid winter wheat. BMC Plant Biol.

[29] Yong-Villalobos L, González-Morales SI, Wrobel K, Gutiérrez-Alanis D, Cervantes-Peréz SA, Hayano-Kanashiro C (2015). Methylome analysis reveals an important role for epigenetic changes in the regulation of the Arabidopsis response to phosphate starvation. Proc Natl Acad Sci USA.

[30] Reed DH, Frankham R (2003). Correlation between Fitness and Genetic Diversity. Conserv Biol.

[31] Guo Z, Li X, He Z, Yang Y, Wang W, Zhong C (2018). Extremely low genetic diversity across mangrove taxa reflects past sea level changes and hints at poor future responses. Glob Change Biol.

[32] Sexton JP, McKay JK, Sala A (2002). Plasticity and Genetic Diversity May Allow Saltcedar to Invade Cold Climates in North America. Ecol Appl.

[33] Frenot Y, Aubry M, Misset MT, Gloaguen JC, Gourret JP, Lebouvier M. Phenotypic plasticity and genetic diversity in Poa annua L. (Poaceae) at Crozet and Kerguelen Islands (subantarctic). Polar Biol. 1999;22:302–10.

[34] Geng Y, van Klinken RD, Sosa A, Li B, Chen J, Xu C-Y. The Relative Importance of Genetic Diversity and Phenotypic Plasticity in Determining Invasion Success of a Clonal Weed in the USA and China. Front Plant Sci. 2016;7. doi:10.3389/fpls.2016.00213. 10.3389/fpls.2016.00213 PMC4764702 26941769

[35] Novaes E, Kirst M, Chiang V, Winter-Sederoff H, Sederoff R (2010). Lignin and Biomass: A Negative Correlation for Wood Formation and Lignin Content in Trees. Plant Physiol.

[36] Huang J, Gu M, Lai Z, Fan B, Shi K, Zhou Y-H (2010). Functional Analysis of the Arabidopsis PAL Gene Family in Plant Growth, Development, and Response to Environmental Stress. Plant Physiol.

[37] Sewalt V, Ni W, Blount JW, Jung HG, Masoud SA, Howles PA (1997). Reduced Lignin Content and Altered Lignin Composition in Transgenic Tobacco Down-Regulated in Expression of L-Phenylalanine Ammonia-Lyase or Cinnamate 4-Hydroxylase. Plant Physiol.

[38] Shafrin F, Das SS, Sanan-Mishra N, Khan H (2015). Artificial miRNA-mediated down-regulation of two monolignoid biosynthetic genes (C3H and F5H) cause reduction in lignin content in jute. Plant Mol Biol.

[39] Elkind Y, Edwards R, Mavandad M, Hedrick SA, Ribak O, Dixon RA (1990). Abnormal plant development and down-regulation of phenylpropanoid biosynthesis in transgenic tobacco containing a heterologous phenylalanine ammonia-lyase gene. PNAS.

[40] Reddy MSS, Chen F, Shadle G, Jackson L, Aljoe H, Dixon RA. Targeted down-regulation of cytochrome P450 enzymes for forage quality improvement in alfalfa (Medicago sativa L.). PNAS. 2005;102:16573–8. 10.1073/pnas.0505749102 PMC1283808 16263933

[41] Ralph J, Lundquist K, Brunow G, Lu F, Kim H, Schatz PF, et al. Lignins : natural polymers from oxidative coupling of 4-hydroxyphenyl-propanoids. Phytochemistry reviews Vol 3 (2004): p 29–60. 2004. https://www.fs.usda.gov/treesearch/pubs/22070. Accessed 19 Apr 2021.

[42] Bonawitz ND, Chapple C (2010). The Genetics of Lignin Biosynthesis: Connecting Genotype to Phenotype. Annu Rev Genet.

[43] Niederhuth CE, Bewick AJ, Ji L, Alabady MS, Kim KD, Li Q (2016). Widespread natural variation of DNA methylation within angiosperms. Genome Biol.

[44] Su Y, Bai X, Yang W, Wang W, Chen Z, Ma J (2018). Single-base-resolution methylomes of Populus euphratica reveal the association between DNA methylation and salt stress. Tree Genet Genomes.

[45] Daccord N, Celton J-M, Linsmith G, Becker C, Choisne N, Schijlen E (2017). High-quality de novo assembly of the apple genome and methylome dynamics of early fruit development. Nat Genet.

[46] Schmitz RJ, Schultz MD, Urich MA, Nery JR, Pelizzola M, Libiger O (2013). Patterns of Population Epigenomic Diversity. Nature.

[47] Takuno S, Ran J-H, Gaut BS (2016). Evolutionary patterns of genic DNA methylation vary across land plants. Nature Plants.

[48] Alonso C, Pérez R, Bazaga P, Herrera CM. Global DNA cytosine methylation as an evolving trait: phylogenetic signal and correlated evolution with genome size in angiosperms. Front Genet. 2015;6. doi:10.3389/fgene.2015.00004. 10.3389/fgene.2015.00004 PMC4310347 25688257

[49] Tuskan GA, DiFazio S, Jansson S, Bohlmann J, Grigoriev I, Hellsten U, et al. The Genome of Black Cottonwood, Populus trichocarpa (Torr. & Gray). Science. 2006;313:1596–604. 10.1126/science.1128691 16973872

[50] Uthup TK, Ravindran M, Bini K, Thakurdas S (2011). Divergent DNA Methylation Patterns Associated with Abiotic Stress in Hevea brasiliensis. Mol Plant.

[51] Liang D, Zhang Z, Wu H, Huang C, Shuai P, Ye C-Y (2014). Single-base-resolution methylomes of populus trichocarpa reveal the association between DNA methylation and drought stress. BMC Genet.

[52] Li X, Zhu J, Hu F, Ge S, Ye M, Xiang H (2012). Single-base resolution maps of cultivated and wild rice methylomes and regulatory roles of DNA methylation in plant gene expression. BMC Genomics.

[53] Gutierrez-Arcelus M, Lappalainen T, Montgomery SB, Buil A, Ongen H, Yurovsky A, et al. Passive and active DNA methylation and the interplay with genetic variation in gene regulation. eLife. 2013;2:e00523. 10.7554/eLife.00523 PMC3673336 23755361

[54] Niederhuth CE, Schmitz RJ. Putting DNA methylation in context: from genomes to gene expression in plants. Biochimica et Biophysica Acta (BBA) - Gene Regulatory Mechanisms. 2017;1860:149–56. 10.1016/j.bbagrm.2016.08.009 PMC5203807 27590871

[55] Kakutani T (1997). Genetic characterization of late-flowering traits induced by DNA hypomethylation mutation in Arabidopsis thaliana. Plant J.

[56] Sheldon CC, Burn JE, Perez PP, Metzger J, Edwards JA, Peacock WJ (1999). The FLF MADS Box Gene: A Repressor of Flowering in Arabidopsis Regulated by Vernalization and Methylation. Plant Cell.

[57] Burn JE, Bagnall DJ, Metzger JD, Dennis ES, Peacock WJ (1993). DNA methylation, vernalization, and the initiation of flowering. PNAS.

[58] Finnegan EJ, Genger RK, Kovac K, Peacock WJ, Dennis ES (1998). DNA methylation and the promotion of flowering by vernalization. PNAS.

[59] Pabón-Mora N, Ambrose BA, Litt A (2012). Poppy APETALA1/FRUITFULL Orthologs Control Flowering Time, Branching, Perianth Identity, and Fruit Development. Plant Physiol.

[60] Seo E, Lee H, Jeon J, Park H, Kim J, Noh Y-S (2009). Crosstalk between Cold Response and Flowering in Arabidopsis Is Mediated through the Flowering-Time Gene SOC1 and Its Upstream Negative Regulator FLC. Plant Cell.

[61] Jung J-H, Ju Y, Seo PJ, Lee J-H, Park C-M (2012). The SOC1-SPL module integrates photoperiod and gibberellic acid signals to control flowering time in Arabidopsis. Plant J.

[62] Lee JH, Park SH, Lee JS, Ahn JH. A conserved role of SHORT VEGETATIVE PHASE (SVP) in controlling flowering time of Brassica plants. Biochimica et Biophysica Acta (BBA) - Gene Structure and Expression. 2007;1769:455–61. 10.1016/j.bbaexp.2007.05.001 17566572

[63] Brill EM, Watson JM (2004). Ectopic expression of a Eucalyptus grandis SVP orthologue alters the flowering time of Arabidopsis thaliana. Functional Plant Biol.

[64] Lee JH, Yoo SJ, Park SH, Hwang I, Lee JS, Ahn JH (2007). Role of SVP in the control of flowering time by ambient temperature in Arabidopsis. Genes Dev.

[65] Kazan K, Lyons R (2016). The link between flowering time and stress tolerance. J Exp Bot.

[66] Richter R, Bastakis E, Schwechheimer C (2013). Cross-Repressive Interactions between SOC1 and the GATAs GNC and GNL/CGA1 in the Control of Greening, Cold Tolerance, and Flowering Time in Arabidopsis. Plant Physiol.

[67] Debieu M, Tang C, Stich B, Sikosek T, Effgen S, Josephs E, et al. Co-Variation between Seed Dormancy, Growth Rate and Flowering Time Changes with Latitude in Arabidopsis thaliana. PLOS ONE. 2013;8:e61075. 10.1371/journal.pone.0061075 PMC3662791 23717385

[68] Khan MNI, Suwa R, Hagihara A. Allometric relationships for estimating the aboveground phytomass and leaf area of mangrove Kandelia candel (L.) Druce trees in the Manko Wetland, Okinawa Island, Japan. Trees-Struct Funct. 2005;19:266–72.

[69] Bolger AM, Lohse M, Usadel B (2014). Trimmomatic: a flexible trimmer for Illumina sequence data. Bioinformatics.

[70] Li H, Durbin R (2009). Fast and accurate short read alignment with Burrows-Wheeler transform. Bioinformatics.

[71] McKenna A, Hanna M, Banks E, Sivachenko A, Cibulskis K, Kernytsky A (2010). The Genome Analysis Toolkit: A MapReduce framework for analyzing next-generation DNA sequencing data. Genome Res.

[72] Lee T-H, Guo H, Wang X, Kim C, Paterson AH (2014). SNPhylo: a pipeline to construct a phylogenetic tree from huge SNP data. BMC Genomics.

[73] Alexander DH, Novembre J, Lange K (2009). Fast model-based estimation of ancestry in unrelated individuals. Genome Res.

[74] Kim D, Langmead B, Salzberg SL (2015). HISAT: a fast spliced aligner with low memory requirements. Nat Methods.

[75] Pertea M, Pertea GM, Antonescu CM, Chang T-C, Mendell JT, Salzberg SL (2015). StringTie enables improved reconstruction of a transcriptome from RNA-seq reads. Nat Biotechnol.

[76] Love MI, Huber W, Anders S (2014). Moderated estimation of fold change and dispersion for RNA-seq data with DESeq2. Genome Biol.

[77] Krueger F, Andrews SR (2011). Bismark: a flexible aligner and methylation caller for Bisulfite-Seq applications. Bioinformatics.

[78] Akalin A, Kormaksson M, Li S, Garrett-Bakelman FE, Figueroa ME, Melnick A (2012). methylKit: a comprehensive R package for the analysis of genome-wide DNA methylation profiles. Genome Biol.

