# Supplementary material for: Multi-omics analyses on Kandelia obovata reveal its response to transplanting and genetic differentiation among populations
Source: BMC Plant Biol. 2021 Jul 19;21:341. doi: 10.1186/s12870-021-03123-1 (PMC8287808; doi:10.1186/s12870-021-03123-1)
Supplement: Supplementary file 1 — Additional file 1. [file 12870_2021_3123_MOESM1_ESM.docx]

**Supplementary material for:**

**Multi-omics analyses on *Kandelia obovata* reveal its response to transplanting and genetic differentiation among populations**

Yuze Zhao, Yifan Zhong, Congting Ye, Pingping Liang, Xiaobao Pan, Yuan-Ye Zhang, Yihui Zhang, Yingjia Shen

**Table of Contents:**

| **Fig. S1** Location of seeding collecting and common gardens. | Page 2 |
| --- | --- |
| **Fig. S2** PCA of transcriptomic profiles of nine individuals. | Page 2 |
| **Fig. S3** Enriched KEGG pathways for DEGs in Nc relative to Cc. | Page 3 |
| **Fig. S4** Enriched KEGG pathways for DEGs in Sc relative to Cc. | Page 3 |
| **Fig. S5** qRT-PCR validation of the expression level of selected genes. | Page 4 |
| **Fig. S6** Enriched KEGG pathways for DMR-genes in Nc relative to Cc. | Page 4 |
| **Fig. S7** Enriched KEGG pathways for DMR-genes in Sc relative to Cc | Page 4 |
| **Fig. S8** Density curves of methylation level of DEGs in Sc. | Page 5 |
| **Fig. S9** Density curves of methylation level of DEGs in Nc. | Page 5 |
| **Table** **S1** Summary information of SNPs from all individuals. | Page 6 |
| **Table** **S2** Down-regulated genes in transplanted individuals. | Page 7 |
| **Table** **S3** The differentially expressed and methylated TFs. | Page 8 |
| **Table** **S4** Differentially expressed and methylated MADS-box genes. | Page 9 |
| **Table S5** The sequences of primers for qRT-PCR. | Page 9 |


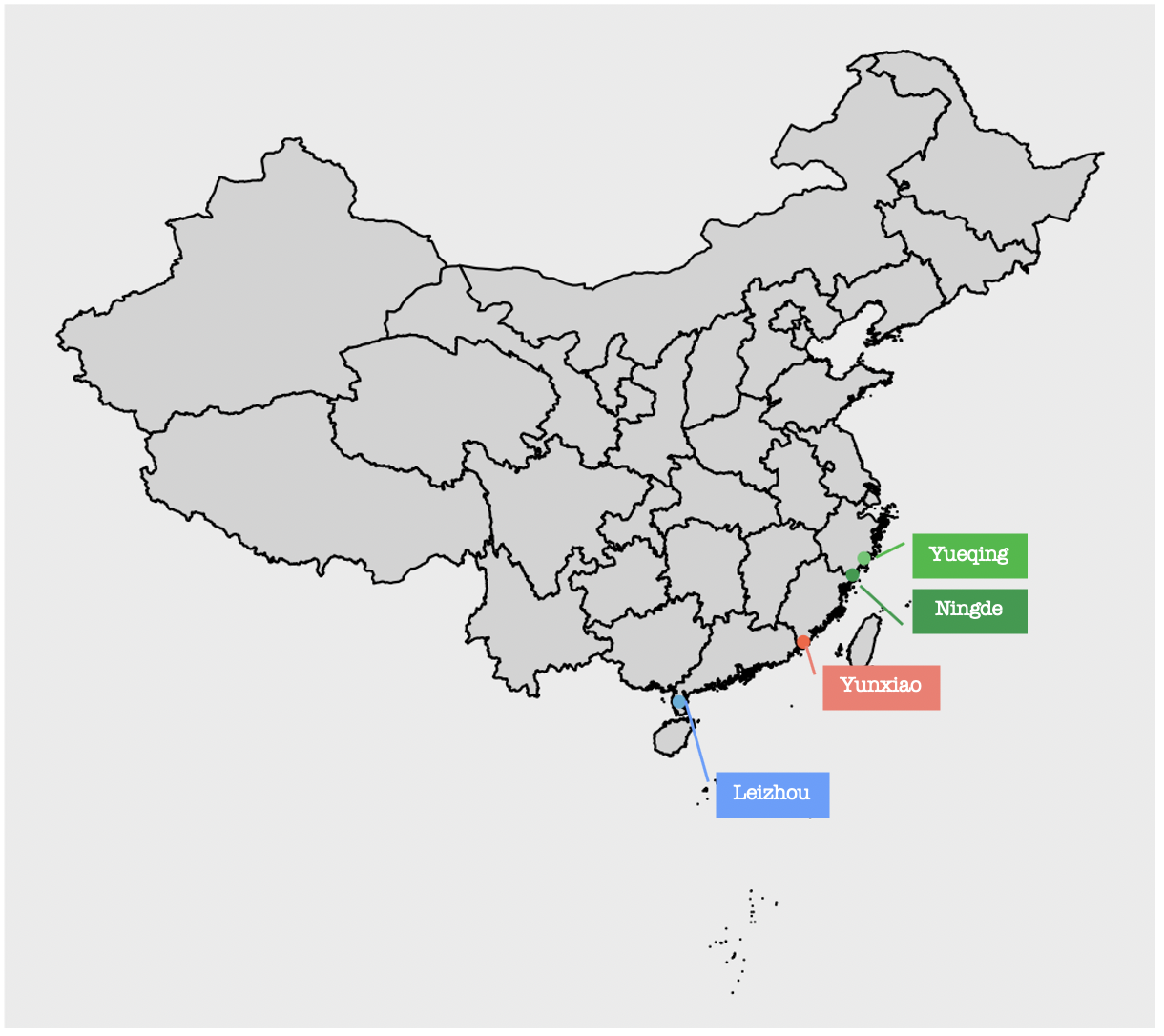


**Fig. S1** Location of seeding collecting and common gardens.

Seedings of *K. obovata* were collected in Leizhou (blue dot), Yunxiao (red dot), and Ningde (dark green dot). Seedings were reciprocally transplanted in the common garden located in Leizhou (blue dot), Yunxiao (red dot), and Yueqing (light green dot).

**
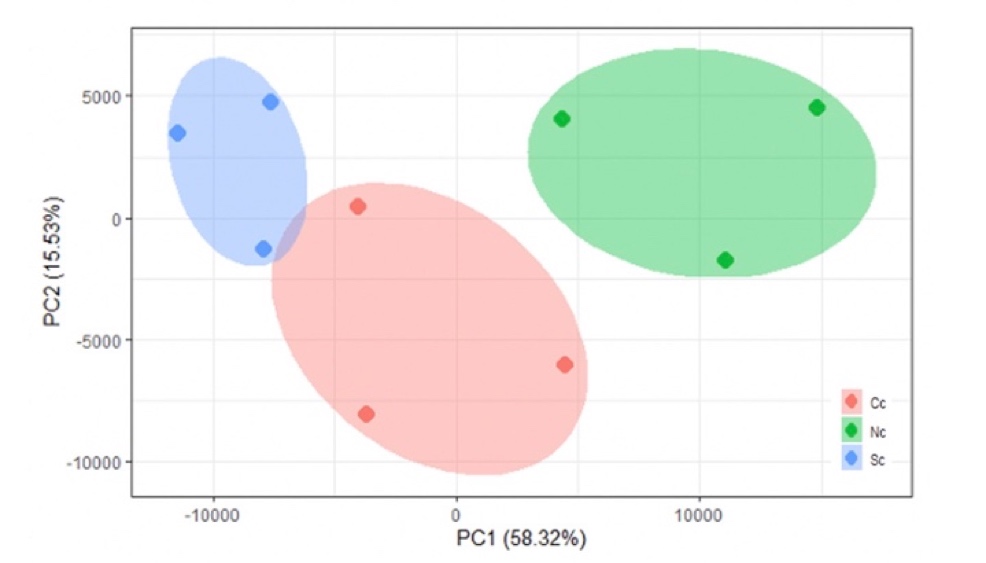
**

**Fig. S2** PCA of transcriptomic profiles of nine individuals.

PCA of transcriptomic profiles of central-origin individuals with three replicates each. Cc: central-origin individuals planted in central garden; Nc: central-origin individuals planted in north garden; Sc: central-origin individuals planted in south garden.


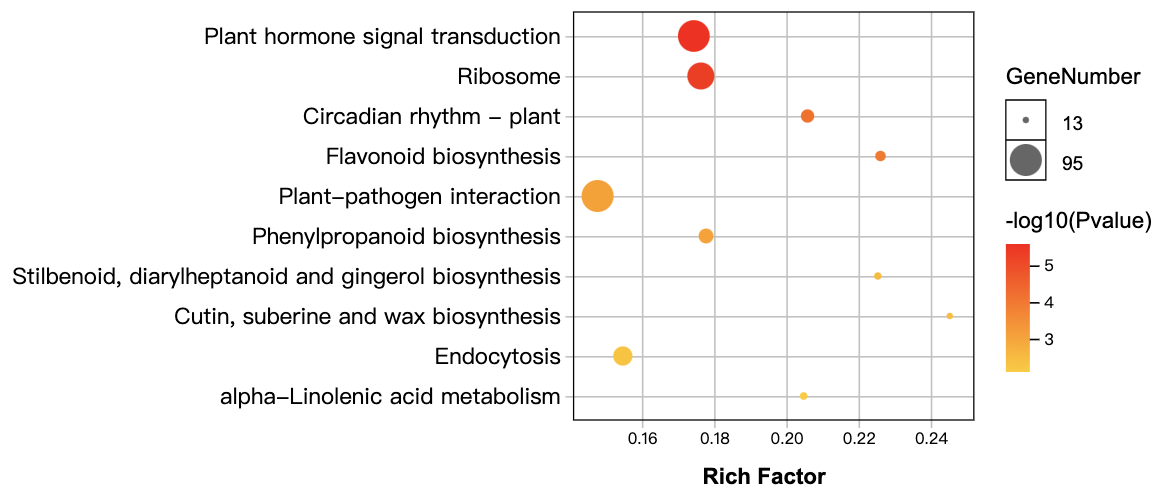


**Fig. S3** Enriched KEGG pathways for DEGs in Nc relative to Cc.

Top ten enriched KEGG pathways for DEGs in Nc (central-origin individuals planted in north garden) relative to Cc (central-origin individuals planted in central garden).


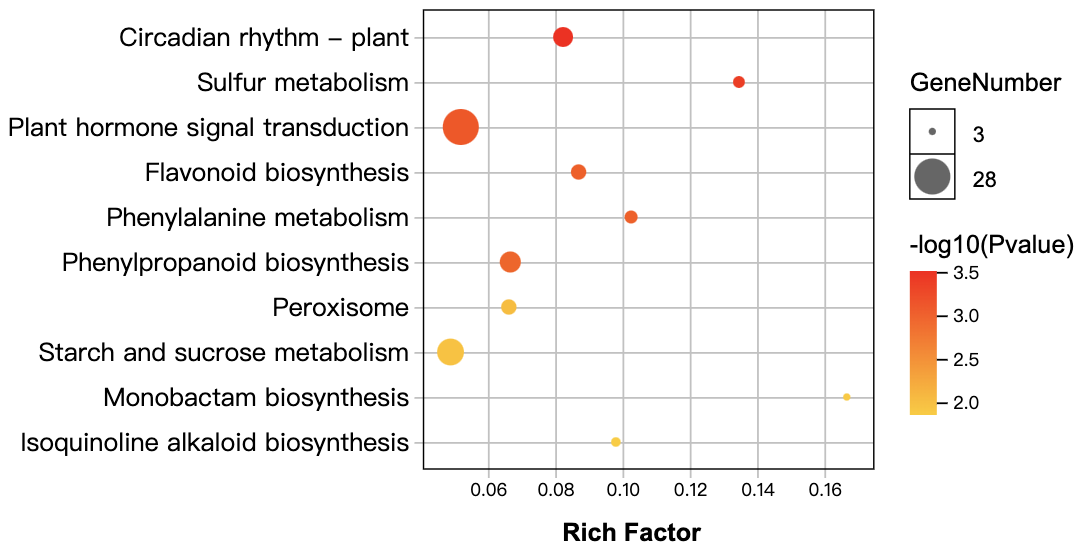


**Fig. S4** Enriched KEGG pathways for DEGs in Sc relative to Cc.

Top ten enriched KEGG pathways for DEGs in Sc (central-origin individuals planted in south garden) relative to Cc (central-origin individuals planted in central garden).


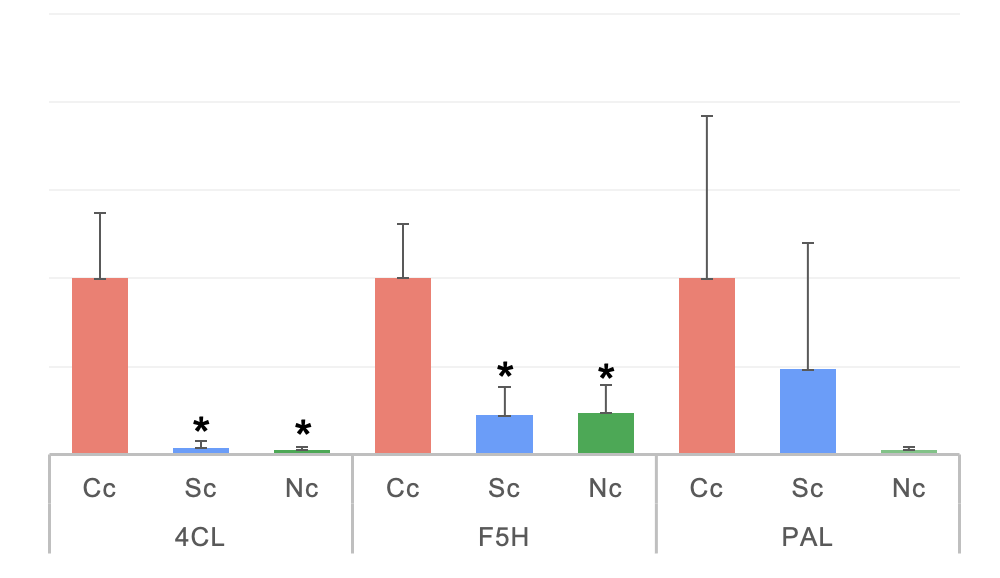


**Fig. S5** qRT-PCR validation of the expression level of selected genes.

Significant differences are shown * (*P* < 0.05)


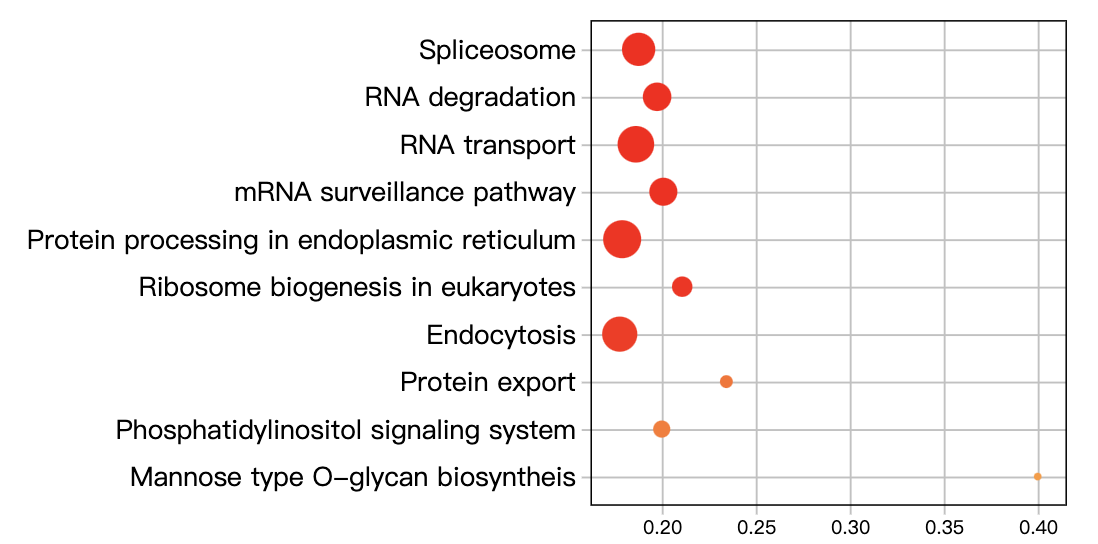


**Fig. S6** Enriched KEGG pathways for DMR-genes in Nc relative to Cc.


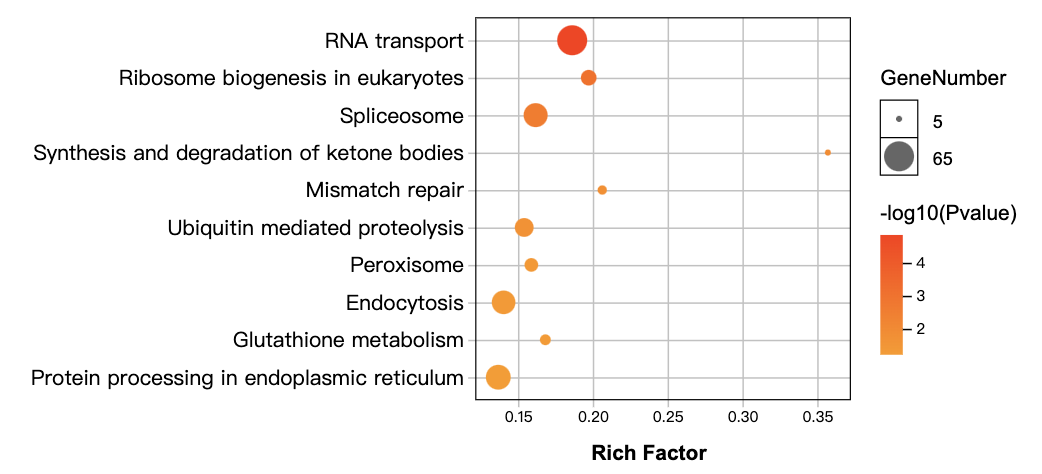


**Fig. S7** Enriched KEGG pathways for DMR-genes in Sc relative to Cc.


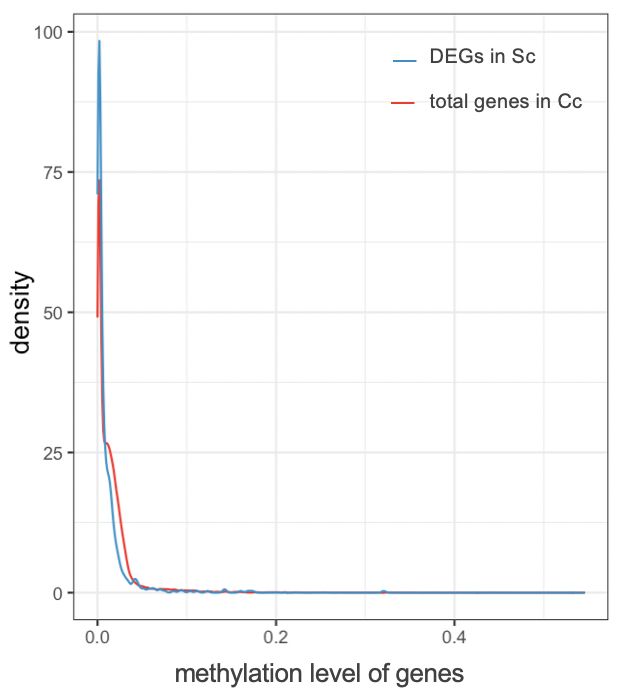


**Fig. S8** Density curves of methylation level of DEGs in Sc.

**
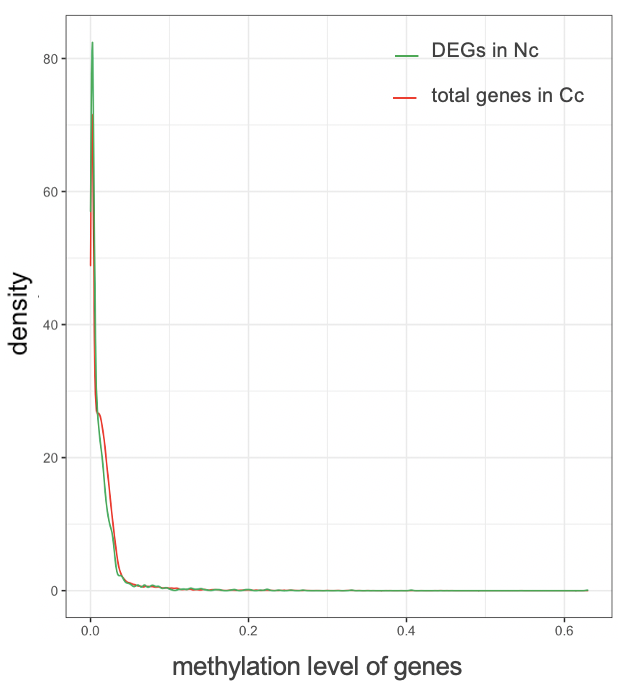
**

**Fig. S9** Density curves of methylation level of DEGs in Nc.

**Table S1** Summary information of SNPs from all individuals mentioned in our study.

| Population | Planting Site | Sample Name | SNPs Number | Homozygous Proportion |
| --- | --- | --- | --- | --- |
| Leizhou  (20°91′N) | Leizhou | Ss_1 | 112414 | 37.61% |
|  |  | Ss_2 | 107460 | 41.17% |
|  | Yunxiao | Cs_1 | 97330 | 54.21% |
|  |  | Cs_2 | 111799 | 39.81% |
|  |  | Cs_3 | 111085 | 38.29% |
| Yunxiao  (23°93′N) | Leizhou | Sc_1 | 96664 | 30.40% |
|  |  | Sc_2 | 93111 | 30.54% |
|  |  | Sc_3 | 91087 | 33.96% |
|  |  | Sc_4 | 91373 | 33.71% |
|  | Yunxiao | Cc_1 | 86819 | 34.49% |
|  |  | Cc_2 | 92249 | 37.73% |
|  |  | Cc_3 | 88593 | 37.15% |
|  | Yueqing | Nc_1 | 94956 | 47.30% |
|  |  | Nc_2 | 94025 | 31.33% |
|  |  | Nc_3 | 95137 | 47.13% |
| Ningde  (27°28′N) | Leizhou | Sn_1 | 86596 | 60.89% |
|  |  | Sn_2 | 84264 | 66.92% |
|  |  | Sn_3 | 85129 | 60.30% |
|  | Yunxiao | Cn_1 | 87107 | 60.21% |
|  |  | Cn_2 | 87480 | 59.39% |
|  |  | Cn_3 | 87825 | 60.19% |
|  | Yueqing  (28°11′N) | Nn_1 | 85545 | 61.02% |

***** The samples marked in yellow were used to study the mechanism of transplantation response.

**Table S2** Down-regulated genes in transplanted individuals (Sc and Nc) and the differences in their expression relative to un-transplanted individuals (Cc).

| Gene Name | Gene ID | log2FC in Sc | log2FC in Nc |
| --- | --- | --- | --- |
| PAL | evm.TU.Contig2685.546 | -1.77 | -1.26 |
| 4CL | evm.TU.Contig2685.995 | -2.13 | -2.42 |
| F5H | evm.TU.Contig11518.11 | -1.77 | -2.03 |

**Table S3** The differentially expressed and methylated transcription factors induced by transplantation.

| geneID | type | name |
| --- | --- | --- |
| evm.TU.Contig11642.1111 | AP2 | WRI1 |
| evm.TU.Contig9220.372 | AP2 | RAP2.7 |
| evm.TU.Contig2581.173 | BES1 | BEH4 |
| evm.TU.Contig9220.71 | bHLH | BHLH60 |
| evm.TU.Contig11642.182 | bZIP | BZIP11 |
| evm.TU.Contig5252.1179 | Dof | CDF2 |
| evm.TU.Contig10660.191 | E2F/DP | E2FF |
| evm.TU.Contig9220.924 | G2-like | EFM |
| evm.TU.Contig2712.546 | GRAS | SHR |
| evm.TU.Contig8624.584 | GRAS | SCL23 |
| evm.TU.Contig5483.278 | HB-PHD | PRH |
| evm.TU.Contig9867.814 | HD-ZIP | HAT5 |
| evm.TU.Contig8573.272 | HD-ZIP | ATHB-13 |
| evm.TU.Contig10143.712 | MIKC_MADS | SVP |
| evm.TU.Contig11642.876 | MIKC_MADS | SVP |
| evm.TU.Contig5386.1069 | MIKC_MADS | SOC1 |
| evm.TU.Contig5386.1291 | MIKC_MADS | SOC1 |
| evm.TU.Contig11642.948 | M-type_MADS | AGL8 |
| evm.TU.Contig14326.548 | M-type_MADS | AGL65 |
| evm.TU.Contig5386.885 | MYB | MYB4 |
| evm.TU.Contig9868.753 | MYB | MYB7 |
| evm.TU.Contig29751.80 | MYB_related | CPC |
| evm.TU.Contig5483.533 | NF-YA | HAP2C |
| evm.TU.Contig2685.177 | SBP | SPL6 |
| evm.TU.Contig2712.1999 | SRS | SRS6 |
| evm.TU.Contig11642.474 | Trihelix | AT3G10030 |
| evm.TU.Contig9220.489 | Trihelix | AT1G76870 |

**Table S4** Differentially expressed and methylated MADS-box genes in transplanted *K. obovata*

| name | gene ID | log2FC | DMR context | hyper(+)/hypo(-) | DMR genetic  location |
| --- | --- | --- | --- | --- | --- |
| FUL | evm.TU.Contig11642.948 | -3.7 | CG | - | Intron |
| SVP | evm.TU.Contig10143.712 | -1.3 | CG | - | Intron |
| SVP | evm.TU.Contig11642.876 | -2.4 | CG | - | Intron |
| SOC1 | evm.TU.Contig5386.1069 | -2.6 | CG | - | Intron |
| SOC1 | evm.TU.Contig5386.1291 | -5.4 | CG | - | Intron |

**Table S5** The sequences of primers for qRT-PCR

| Primer | sequence (5'to3') |
| --- | --- |
| PAL-F | CATAGAAGAACCAAGCAAGG |
| PAL-R | GCAATGTGTGGCAAGATT |
| 4CL-F | CTGCCTAACGCTAAACTTG |
| 4CL-R | ACGCTCCTGACTTAATCTC |
| F5H-F | TCCAGAACTCCATCAAACT |
| F5H-R | GCCCACTCTATCACAGAT |
